# Evaluating Shared Decision-Making in Postpartum Contraceptive Counseling Using Objective Structured Clinical Examinations

**DOI:** 10.1089/whr.2022.0067

**Published:** 2022-12-26

**Authors:** Brownsyne Tucker Edmonds, Shelley M. Hoffman, Tatiana Laitano, Fatima McKenzie, Janet Panoch, Abigail Litwiller, Mark J. DiCorcia

**Affiliations:** ^1^ Department of Obstetrics and Gynecology, Indiana University School of Medicine, Indianapolis, Indiana, USA.; ^2^ Department of Obstetrics and Gynecology, University of Illinois College of Medicine, Chicago, Illinois, USA.; ^3^ Integrated Medical Science Department, Florida Atlantic University Charles E. Schmidt College of Medicine, Boca Raton, Florida, USA.

**Keywords:** postpartum contraception, counseling, shared decision-making, communication

## Abstract

**Background:**

Shared decision-making (SDM) may support widespread uptake of progestin-containing long-acting reversible contraceptives in the immediate postpartum period. We piloted an Objective Structured Clinical Examination (OSCE) to evaluate first-year obstetrics and gynecology resident physicians' use of SDM in postpartum contraception counseling.

**Methods:**

As part of their 2015 and 2016 OSCEs, first-year OB/GYN residents were instructed to provide contraceptive counseling to a Standardized Patient (SP) portraying a 29-year-old postpartum patient seen during rounds on the morning following her delivery. Three investigators independently scored each resident encounter using a 10-item rubric adapted from a 9-item SDM measure and assigned scores of 0 (absent), 1 (partial), or 2 (complete). Each encounter was video and audio recorded, then transcribed for qualitative analysis. Descriptive statistics was produced using SPSS version 24.

**Results:**

Eighteen residents participated. The majority (78%) discussed contraceptive options and timing of initiation. Nearly 33% elicited factors most important to the SP in influencing her preference. Only 6% discussed the benefits of exclusive breastfeeding, and few addressed the uncertainty of progesterone on milk supply and production.

**Conclusion:**

Although residents conveyed ample clinical information, the vast majority did not discuss elements of SDM, such as her preferences, values, and goals for future fertility and breastfeeding. Our work revealed that critical elements of SDM are often not explored and deliberated by resident physicians. Trainings (*e.g.*, OSCEs) are needed to equip residents with effective communication skills to facilitate more SDM in postpartum contraceptive care.

## Introduction

Achieving optimal birth spacing is necessary for preventing short interval pregnancies, or pregnancies within 18 months from the previous delivery, which may result in adverse maternal and perinatal outcomes, such as low birth weight, preterm premature rupture of the membranes, and developmental disorders. In the United States alone, half of all pregnancies are likely to be unintentional, and one-third of women experience short-interval pregnancies.^1,2^ Long-acting reversible contraception (LARC), including intrauterine devices and the etonogestrel implant, offers women a safe and effective way to achieve adequate birth spacing and can be initiated immediately postpartum before hospital discharge.^3,4^ Immediate or early postpartum LARC may reduce short interval pregnancy,^5^ especially for women who face barriers to attending postpartum visits^6,7^ or accessing care outside of pregnancy.^8–10^

Choosing a contraceptive method in the postpartum period can sometimes conflict with other important goals for maternal and infant health, including the duration and exclusivity of breastfeeding. In 2017, the U.S. infant mortality rate was 5.8 deaths per 1000 live births and was highest among black women at 11.4 deaths per 1000 live births compared to white women at 4.9 per 1000.^11^ Although the causes of infant mortality are multifactorial, two well-documented strategies to reduce infant mortality and morbidity are achieving adequate birth spacing and exclusive breastfeeding for at least 6 months.^12–15^ As more effective contraceptive methods are developed and become available to patients, the decision to choose a method that is both effective and safe during breastfeeding has become an increasingly complex yet important discussion between a patient and her provider.

However, there is limited research that explores how breastfeeding women navigate the decision for a postpartum contraceptive method. A study by Loewenberg Weisband et al found that breastfeeding participants surveyed before hospital discharge had high intentions to use contraception. Of these, only 21% of the participants considered the safety of contraception on the mother's and infant(s) health.^16^ Moreover, the most common reason for their preferred choice of contraception was convenience.

Conversations with health care providers about the risks, benefits, alternatives, and affordability of different contraception methods should begin early in pregnancy and continue in the postpartum period.^17^ Recent surveys indicate that many women do not feel knowledgeable nor have in-depth consultations with their providers to discuss aspects of contraceptive use, such as which options are available, the benefits and risks associated with each option, as well as their personal values and goals in birth control, all critical factors in making an informed decision about the best method that would support their desires for breastfeeding and birth spacing.^12–13^

American College of Obstetricians and Gynecologists (ACOG) recommends giving breastfeeding women progestin-containing contraception in the immediate postpartum period, as opposed to combined hormonal methods, which may place some women at greater risk for venous thromboembolism.^18^ Because milk production is believed to be triggered by the reduction of progesterone that results from placental removal at delivery, there is a theoretical risk that introducing progestin after delivery may inhibit this process. However, evidence from clinical trials and observational studies of immediate postpartum progestin-containing contraception have not demonstrated adverse breastfeeding outcomes.^19,20^ As such, the U.S. Medical Eligibility Criteria (US MEC) for Contraceptive Use recognize that the benefits of using progestin-containing contraception in the immediate postpartum period far outweigh the theoretical risks.^21^

Interpretation of this theoretical risk has varied between health care providers, specifically obstetricians and lactation consultants.^22–24^ In a study by Dunn et al. with lactation providers, 93% of respondents felt that the theoretical negative impacts that progestin may have on breastfeeding within the early postpartum period outweighed the benefits, despite the US MEC guidelines.^23^ Because they interact with a multitude of health care providers, postpartum patients may potentially receive inconsistent guidance regarding LARC impact on breastfeeding.^22^ In some circumstances, breastfeeding patients may be discouraged to obtain progestin-containing LARC in the immediate postpartum period, placing those who are unable to follow-up for care at greater risk for short interval pregnancies.

This conflicting information can prevent women from making well-informed confident decisions about their reproductive health, and given that both birth spacing and exclusive breastfeeding mitigate risks of adverse maternal and child health outcomes, these decisions are critically important.

Shared decision-making (SDM) is an optimal approach to enhancing patient-centered communications^25^ about postpartum contraception and can support women in deciding which options most align with their contraceptive preferences and goals for breastfeeding and birth spacing.^22,26^ SDM is a framework that encourages providers to comprehensively counsel patients on all options, along with the risks and benefits, and incorporate the patient's preferences, values, and goals into the clinical decision-making.^27^ Rather than lactation consultants and obstetricians pursuing their own agendas and priorities, postpartum contraception counseling should focus on identifying the woman's desires for future pregnancies, exclusive breastfeeding, and the method of contraception that best fits her lifestyle.^28,29^ An SDM approach would center on eliciting what the patient prioritizes and helping her identify the contraceptive method that would best allow her to achieve those goals.

Current Accreditation Council for Graduate Medical Education Milestones^30,31^ require that obstetrics and gynecology (OBGYN) resident physicians demonstrate competency in SDM. Objective structured clinical examinations (OSCEs) are widely used in medical residencies to evaluate both clinical and communication skills and can be a useful tool for measuring residents' use of SDM. We created an OSCE to assess and train residents on postpartum contraceptive counseling. In this study, we present the results of this assessment activity, which sought to develop and pilot test an OSCE to evaluate the use of SDM in counseling for postpartum contraception among postgraduate first-year (PGY-1) OBGYN physician residents.

## Methods

### Study design

We conducted an exploratory single-center simulation study in 2015 and 2016 to assess postpartum contraception counseling among OBGYN residents with a specific focus on utilizing SDM to elicit patient preferences, priorities, and concerns surrounding her interest in postpartum contraception. We developed the OSCE for PGY-1 residents because they commonly conduct postpartum rounds in training programs. We were particularly interested in how PGY-1 residents respond to patient concerns regarding progestin-containing LARC and safety related to exclusively breastfeeding. First year residents complete the OSCE in March or April of each year. By this time, all but two of the residents will have completed rotations of L&D and participated in postpartum rounding. By this time of the year, all residents would have been introduced to didactic content on contraceptive counseling.

This OSCE study was piloted among our institution's annual OBGYN resident OSCEs and took place at our university's medical simulation center. While all residents were required to complete the OSCE, inclusion of their data into this study was voluntary. This study was approved by the Indiana University Institutional Review Board (IRB No. 1304011110), and residents were consented verbally.

### The case

Residents were presented with Regina Wilson, a healthy 29-year-old female, who is 1-day postpartum and is interested in discussing her options for postpartum contraception with the resident during their morning inpatient rounds. She just delivered her third daughter and did not experience any labor complications. Ms. Wilson is unsure of which birth control option(s) will enable her to carry out her goal of exclusively breastfeeding for at least 6 months. However, she struggled with milk production during her previous pregnancy. Although she is not opposed to having another child in the future, Ms. Wilson does not desire to be pregnant for the next few years. In addition, she would prefer to avoid using oral contraceptive pills and does not want to get a shot every three months. If asked which is most important to her—birth control or breastfeeding—she would say that successfully breastfeeding is her most immediate priority.

Since the university's Simulation Center provides trained and experienced Standardized Patients (SPs) to implement OSCEs, the study team did not have to recruit or train the SP for this particular study. Instead, the professional SP was provided information before the start of the OSCE regarding her character's psychosocial profile and specific instructions on a standardized set of responses or decisions to make based on the contraceptive options, risks, and benefits presented by the resident (Supplementary Appendix SA1). For instance, if the resident informed Ms. Wilson that progestin-containing contraceptives could potentially harm her chances of being able to breastfeed, the SP was instructed to postpone contraception until a later time. However, if presented with risks and benefits associated with exclusive breastfeeding and short-interval pregnancy, along with an explanation that, despite the theoretical risk, most studies have not identified harmful effects from progestin, then the SP would opt to have the implant before being discharged from the hospital.

### Trainee instructions

Residents reviewed a door note before the start of the examination, which included the patient's name, complaint, history of present illness, and a physical examination report (Supplementary Appendix SA2). The case did not require residents to use any specific materials or equipment. Upon being prompted to start the encounter, the residents had ∼15 minutes to counsel the SP as they would in routine practice and develop a plan of care for her contraceptive needs.

### Coding

A 10-item scoring rubric was adapted from Braddock et al.,^32–34^ who created a 9-item measure to assess the use of shared decision-making in patient–provider communications (Supplementary Appendix SA3). Discussion of the pros and cons is assessed in one item on the original scale. For purposes of this study, we expanded that item into two separate items, specifically to assess residents' discussions of the risks (item 4a) and benefits (item 4b) associated with the multitude of contraceptive methods that can be presented to the SP. A similar version of this codebook has been used in other OSCEs at our institution to assess the use of SDM in discussions pertaining to periviable delivery^35^ and vaginal birth after cesarean.^36^ Each item was assigned a score of absent (0 points), partial (1 point), or complete (2 points).

To receive a “complete” score for each of the 9 items, residents had to address the following: Item 1: a shared decision-making role, Item 2: both sides of the clinical issue and nature of the decision, Item 3: present at least two immediate postpartum contraceptive options, as well as the option to delay initiation until the postpartum visit, Item 4a: discuss at least one risk for each option presented, Item 4b: discuss one benefit for each option presented, Item 5: elicit at least two goals that the patient may have surrounding breastfeeding, contraception, and/or future fertility, Item 6: address uncertainties related to the impact of progestin-containing contraceptives on breastfeeding and failure rates associated with contraception, Item 7: inquire whether the patient wants input from others in the decision-making process, Item 8: assess the patient's understanding of the consultation using teach-back or Ask-Tell-Ask methods, and finally, Item 9: explore the patient's preferences.

If an item was not mentioned or addressed at all, it was counted as “absent” (0 points), and if the topic was addressed, but did not meet the “complete” standard, then “partial” credit was assigned (1 point). Representative quotes of “absent,” “partial,” and “complete” are provided for each element in Supplementary Appendix SA3. An additional five items were scored dichotomously (yes/no) based on the resident's discussion of the SP's priorities (*i.e.*, breastfeeding or contraception), the benefits of breastfeeding, the theoretical risks that progestin-containing contraceptives pose to breastmilk supply and production, the risks associated with short interval pregnancies, and the importance of protecting against sexually transmitted infections (STIs).

Three trained members of the research team (B.T.E., F.M., J.P.) independently scored each patient encounter, and discrepancies were resolved through consensus. Each resident encounter was audio and video recorded and transcribed verbatim. Independent scores and consensus scores were stored in REDCap. Frequencies and descriptive statistics were analyzed using SPSS version 24.

## Results

**FIG. 1.**
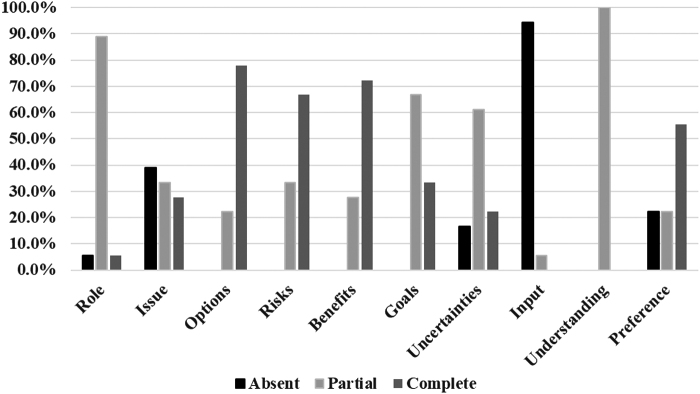
Resident rubric consensus scores for the postpartum OSCE (2015 and 2016). OSCE, objective structured clinical examination.

A total of 18 PGY-1 residents participated in the OSCE (2015 = 10; 2016 = 8), and all agreed to have their data included in this study. Most residents were female (88.9%) and white (72.2%). Their average age was 30 years (range: 27–41 years) (Table 1). Table 2 lists the mean and median scores for all rubric items. The majority (88.9%) received partial credit for indicating that the patient has a role in the decision-making process, but only one resident alluded to a shared decision-making process (Fig. 1). When discussing the clinical issues, roughly one-quarter (27.8%) of residents addressed both issues of future pregnancy and effects of contraception on lactation. The majority (77.8%) received complete scores for presenting multiple contraceptive options. All residents informed the patient of progestin-only and nonhormonal options, and 94.4% presented combined-hormonal contraception as an alternative. Two-thirds (66.7%) received complete scores for discussing the risks, and over 72.2% discussed the benefits of each alternative.

**Table 1.** Obstetrics and Gynecology Postgraduate First-Year Resident Sociodemographics (*N* = 18)

Characteristics	*N* (%)
Age, years, mean (range)	30 (27–41)
Sex	
Male	2 (11.1)
Female	16 (88.9)
Race	
White or European American	13 (72.2)
African American or Black	2 (11.1)
Asian or Pacific Islander	2 (11.1)
Latin American or Hispanic	1 (5.6)
Year of objective structured clinical examination	
2015	10 (55.6)
2016	8 (44.4)

**Table 2.** Median and Mean Values of Postgraduate First-Year Residents' Objective Structured Clinical Examination Rubric Scores

Rubric item	Median score	Mean score
1. Discussion of the patient's role in decision-making	1	1.00
2. Discussion of the clinical issue or nature of the decision	1	0.89
3. Does the resident discuss alternatives (overall score)	2	1.78
a. Explicit discussion of the following options		
i. Combined hormonal	1	0.94
ii. Progesterone	1	1.00
iii. Nonhormonal	1	1.00
b. Explicit discussion of delayed initiation	1	0.78
4a. Does the resident discuss risks of the methods	2	1.67
4b. Does the resident discuss benefits of the methods	2	1.17
5. Does the resident discuss the patient's goal/context of decision?	1	1.33
6. Does the resident discuss the uncertainties associated with the decision?	1	1.06
7. Does the resident discuss the patient's desire for input from others?	0	0.06
8. Does the resident assess the patient's understanding?	1	0.06
9. Does the resident explore the patient's preferences?	2	1.00

One-third (33.3%) of residents received a complete score, while the remaining 66.7% received partial credit, for addressing the patient's goals/context in her decision-making. Furthermore, 22.2% of residents addressed the uncertainties surrounding both the possible impact of contraception on breastfeeding (*i.e.*, milk supply, production) and the failure rates associated with contraception. Only one resident explicitly asked if the patient wanted to consult with her partner or a member of her family regarding her decision, and approximately half of the residents (55.6%) elicited her contraceptive preferences. All residents received partial scores for inviting questions, and none used techniques such as “ask-tell-ask” or “teach back” to receive a complete score in assessing the patient's understanding.

While nearly 72.2% of residents addressed the impact of contraceptive options on breastfeeding, the vast majority failed to assess the patient's priority for successful breastfeeding versus effective contraception (94.4%) and the benefits of exclusive breastfeeding (94.4%). In addition, 88.9% did not inform the patient of the potential maternal and fetal risks associated with short interval pregnancies. Only three residents (16.7%) discussed the need for additional protection from STIs if the patient opted for a nonbarrier contraceptive method.

## Discussion

We set out to develop and pilot test an OSCE to evaluate the use of SDM in postpartum contraception counseling by PGY-1 OBGYN residents. We found that residents successfully addressed the clinical components of postpartum contraception counseling (*e.g.*, options, risks, and benefits), but often failed to utilize important elements of SDM to identify the method that best aligned with the patient's preferences and goals. In particular, the vast majority of residents failed to explore the patient's breastfeeding intentions and discuss the uncertainties surrounding breast milk supply and production, thereby missing the opportunity to put into context the theoretical risk of progestins on lactation alongside the competing risks related to short-interval pregnancy.

SDM requires a level of partnership, negotiation, and shared power between patients and providers.^37,38^ It requires that providers recognize that patients bring expertise about their unique context and lived experience to bear on their own clinical decision-making. In preference sensitive decision-making, in which the task at hand is not to identify what's “right,” but rather, “what's right for you” in the patient encounter, a provider should not be able to make treatment “recommendations” in the absence of adequately engaging the patient in an exchange that explores their goals and elicits their preferences to inform that recommendation.^39^

In the case of postpartum contraception there is no one “right” answer for every patient. Nevertheless, providers may apply a “one-size fits all spiel” to contraceptive counseling that fails to consider the knowledge, experience, or needs of an individual patient in their approach.^40^ Appropriate counseling requires understanding a number of factors related to patient's intentions for pregnancy, desires to pursue or avoid pregnancy, desired timing and/or spacing of pregnancies, prior experiences with contraception, personal preferences, knowledge, and fears. A myriad of factors may inform the optimal contraceptive method for any given patient at a given time.^41^

In the postpartum setting, these considerations are further complicated by the issue of breastfeeding, which has long been a source of debate and controversy between breastfeeding advocates and the contraceptive community. Despite the lack of evidence of harm or detriment to milk supply from progestin-only contraceptive methods, the theoretical risk continues to loom large in the minds of many breastfeeding women.^42–44^ Some may be unwilling to take any chance, even if theoretical, to jeopardize their chances at breastfeeding success if they are counseled by lactation consultants that contraception may pose a threat.

With this in mind, the conversation regarding immediate postpartum progestin-containing LARC becomes particularly fraught and requires skilled engagement to determine how a given patient may weigh competing priorities, tolerate risks for unintended pregnancy or short-interval pregnancy, or assign exclusive breastfeeding as her primary goal or concern. Given this complexity, obstetrical providers are in need of training and tools to master effective approaches to engage in these encounters in a manner that is patient centered rather than proscriptive or generic. OSCEs provide an opportunity to apply what is learned in didactic lectures and during patient encounters with observation of attendings and more senior residents. Our OSCE provides an initial tool for assessing these advanced communication skills among trainees.

Major concerns have arisen in the reproductive justice community regarding the potential for coercion in contraceptive counseling, targeting women of color, low-income women, and substance-using women for LARC utilization based on perceptions of “fitness” for reproduction and/or motherhood.^45–47^ Providers carry biases, both implicit and explicit, that impact their counseling approach and messaging.^40^ SDM holds some promise in mitigating such biases by centering the patient's goals and preferences rather than provider's presumptions about what will serve in a given woman's “best interest.” Training in SDM in contraceptive counseling may help to promote more shared, informed decision-making regarding patients' desired reproductive lives and family plans because the framework supports aligning treatment recommendations with patients' goals of care. In future studies, this OSCE could be used to longitudinally evaluate PGYs as they advance through residency to determine if their SDM skills improve or grow. In addition, our findings could be utilized to inform intervention development or curricular components to help improve OSCE scores throughout the years.

In interpreting our findings, we could observe various limitations. The study was performed in a single center, assessing first-year OBGYN residents’, which makes it difficult to generalize our results to different populations. Our results could be skewed by Hawthorne effects and social desirability biases, seeing as the residents may provide more comprehensive SDM counseling and strive to demonstrate more socially desirable responses and behaviors toward the SP when they know that the patient encounter is being recorded as part of the OSCE. In an effort to mitigate social desirability bias, we did not inform residents of our SDM assessment or use of SDM for patient counseling in advance. Moreover, resident performance might not have been an accurate representation of their actual counseling behaviors when in practice, and there may be overestimations of their SDM behaviors in our results.

We also acknowledge that although SPs are thoroughly trained for OSCEs, SPs cannot simulate all possible clinical situations. Consequently, in the actual postpartum inpatient encounter, residents may face a patient with an unexpected or unusual clinical circumstance and who may be more overwhelmed, sleep deprived, and tired than the SPs they encountered. Therefore, when in practice, residents might perform poorer than in their OSCEs,^48^ and unpredictable real-life encounters may require deeper communication skills by doctors to elicit their patient's values. Despite the aforementioned limitations that come with simulated settings with SPs, OSCE testing continuously demonstrates to be a powerful tool for medical staff performance outcome and evaluation.^49^

## Conclusion

In our study, OB/GYN residents consistently conveyed ample medical information, but often failed to address the patients' goals influencing her contraceptive preference and uncertainties regarding breastfeeding implications. This suggests that critical elements of SDM are not often explored and deliberated. The safety of progestin containing LARC methods for breastfeeding women remains contested in the lactation community. This serves as a barrier to promoting more widespread uptake of LARC methods in the immediate postpartum period. SDM may offer a strategy to negotiate this divide. Interventions and resident physician training are needed to facilitate more shared, informed decision-making around postpartum contraceptive care and improve communication skills that are critical to SDM.

## Supplementary Material

Supplemental data

Supplemental data

Supplemental data

## Supplementary Material

Supplementary Appendix SA1

Supplementary Appendix SA2

Supplementary Appendix SA3

## Abbreviations Used

LARC: long-acting reversible contraception
OBGYN: obstetrics and gynecology
OSCE: objective structured clinical examination
PGY-1: postgraduate first-year
SDM: shared decision-making
SP: standardized patient
STIs: sexually transmitted infections
US MEC: U.S. medical eligibility criteria

## References

[1] Gemmill A, Lindberg LD. Short interpregnancy intervals in the United States. Obstet Gynecol 2013;122:64–71. 23743455 10.1097/AOG.0b013e3182955e58 PMC3749871

[2] Cheslack Postava K, Winter AS. Short and long interpregnancy intervals: Correlates and variations by pregnancy timing among U.S. women. Perspect Sex Reprod Health 2015;47:19–26. 25623196 10.1363/47e2615

[3] Henderson V, Stumbras K, Caskey R, Haider S, Rankin K, Handler A. Understanding factors associated with postpartum visit attendance and contraception choices: Listening to low-income postpartum women and health care providers. Matern Child Health J 2016;20(Suppl 1):132–143. 27342600 10.1007/s10995-016-2044-7 PMC5290059

[4] Moniz M, Chang T, Heisler M, Dalton VK. Immediate postpartum long-acting reversible contraception: The time is now. Contraception 2017;95:335–338. 27913231 10.1016/j.contraception.2016.11.007 PMC5453506

[5] Wu M, Eisenberg R, Negassa A, Levi E. Associations between immediate postpartum long-acting reversible contraception and short interpregnancy intervals. Contraception 2020;102:409–413. 32918870 10.1016/j.contraception.2020.08.016

[6] Schwandt HM, Skinner J, Hebert LE, Cobb L, Saad A, Odeku M. Inadequate birth spacing is perceived as riskier than all family planning methods, except sterilization and abortion, in a qualitative study among urban Nigerians. BMC Womens Health 2017;17:80–80. 28893235 10.1186/s12905-017-0439-2 PMC5594467

[7] Exavery A, Mrema S, Shamte A, et al. Levels and correlates of non-adherence to WHO recommended inter-birth intervals in Rufiji, Tanzania. BMC Pregnancy Childbirth 2012;12:152–152. 23237623 10.1186/1471-2393-12-152 PMC3573999

[8] Ross J, Hardee K. Access to contraceptive methods and prevalence of use. J Biosoc Sci 2013;45:761–778. 23151399 10.1017/S0021932012000715 PMC3785174

[9] Satterwhite CL, French V, Allison M, Honderick T, Ramaswamy M. Access to contraception in local health departments, four Midwest states, 2017–2018. Contraception 2019;99:363–367. 30871935 10.1016/j.contraception.2019.02.009 PMC6548685

[10] Blanco C. [Access to birth control: A world estimate]. Profamilia 1988;4:17–24. 12281360

[11] Murphy SL XJ, Kochanek KD, Arias E. Mortality in the United States, 2017. NCHS Data Brief; no 328. Hyattsville, MD: National Center for Health Statistics, 2018. 30500322

[12] American Academy of Pediatrics Section on Breastfeeding. Breastfeeding and the use of human milk. Pediatrics 2012;129:e827.22371471

[13] Perera PJ, Ranathunga N, Fernando MP, Sampath W, Samaranayake GB. Actual exclusive breastfeeding rates and determinants among a cohort of children living in Gampaha district Sri Lanka: A prospective observational study. Int Breastfeed J 2012;7:21. 23259860 10.1186/1746-4358-7-21 PMC3546863

[14] Lewycka S, Mwansambo C, Kazembe P, et al. A cluster randomised controlled trial of the community effectiveness of two interventions in rural Malawi to improve health care and to reduce maternal, newborn and infant mortality. Trials 2010;11:88–88. 20849613 10.1186/1745-6215-11-88 PMC2949851

[15] Fotso JC, Cleland J, Mberu B, Mutua M, Elungata P. Birth spacing and child mortality: An analysis of prospective data from the Nairobi urban health and demographic surveillance system. J Biosoc Sci 2013;45:779–798. 22958417 10.1017/S0021932012000570 PMC3785173

[16] Loewenberg Weisband Y, Keder LM, Keim SA, Gallo MF. Postpartum intentions on contraception use and Method choice among breastfeeding women attending a university hospital in Ohio: A cross-sectional study. Reprod Health 2017;14:45. 28320478 10.1186/s12978-017-0307-4 PMC5360022

[17] Pieh Holder KL. Contraception and breastfeeding. Clin Obstet Gynecol 2015;58:928–935. 26457854 10.1097/GRF.0000000000000157

[18] ACOG Practice Bulletin No. 206: Use of hormonal contraception in women with coexisting medical conditions [published correction appears in Obstet Gynecol 2019 Jun;133(6):1288]. Obstet Gynecol 2019;133:e128–e150. 30681544 10.1097/AOG.0000000000003072

[19] Phillips SJ, Tepper NK, Kapp N, Nanda K, Temmerman M, Curtis KM. Progestogen-only contraceptive use among breastfeeding women: A systematic review. Contraception 2016;94:226–252. 26410174 10.1016/j.contraception.2015.09.010 PMC11376434

[20] Kapp N, Curtis K, Nanda K. Progestogen-only contraceptive use among breastfeeding women: A systematic review. Contraception 2010;82:17–37. 20682140 10.1016/j.contraception.2010.02.002

[21] US medical eligibility criteria (US MEC) for Contraceptive USE, 2016. Centers for Disease Control and Prevention. 2021. Available at: https://www.cdc.gov/reproductivehealth/contraception/mmwr/mec/summary.html Accessed August 5, 2021.

[22] Bryant AG, Lyerly AD, DeVane-Johnson S, Kistler CE, Stuebe AM. Hormonal contraception, breastfeeding and bedside advocacy: The case for patient-centered care. Contraception 2019;99:73–76. 30423320 10.1016/j.contraception.2018.10.011

[23] Dunn K, Bayer LL, Mody SK. Postpartum contraception: An exploratory study of lactation consultants' knowledge and practices. Contraception 2016;94:87–92. 26996737 10.1016/j.contraception.2016.03.007 PMC4884468

[24] Berens P, Labbok M; Academy of Breastfeeding Medicine. ABM Clinical Protocol #13: Contraception During Breastfeeding, Revised 2015. Breastfeed Med 2015;10:3–12. 25551519 10.1089/bfm.2015.9999

[25] Dehlendorf C, Henderson JT, Vittinghoff E, et al. Association of the quality of interpersonal care during family planning counseling with contraceptive use. Am J Obstet Gynecol 2016;215:78.e1–e78.e789. 10.1016/j.ajog.2016.01.173 26827879

[26] ACOG Committee Opinion, Number 736, Optimizing Postpartum Care. American College of Obstetricians and Gynecologists. 2018. Available at: https://www.acog.org/clinical/clinical-guidance/committee-opinion/articles/2018/05/optimizing-postpartum-care# Accessed August 5, 2021.

[27] Charles C, Gafni A, Whelan T. Decision-making in the physician–patient encounter: Revisiting the shared treatment decision-making model. Soc Sci Med 1999;49:651–661. 10452420 10.1016/s0277-9536(99)00145-8

[28] Fox E, Reyna A, Malcolm NM, et al. Client preferences for contraceptive counseling: A systematic review. Am J Prev Med 2018;55:691–702. 30342632 10.1016/j.amepre.2018.06.006 PMC6655529

[29] Dehlendorf C, Levy K, Kelley A, Grumbach K, Steinauer J. Women's preferences for contraceptive counseling and decision making. Contraception 2013;88:250–256. 23177265 10.1016/j.contraception.2012.10.012 PMC4026257

[30] Bienstock JL, Edgar L, McAlister R. Obstetrics and gynecology milestones. J Grad Med Educ 2014;6(1 Suppl 1):126–128. 24701275 10.4300/JGME-06-01s1-08 PMC3966600

[31] The obstetrics and gynecology milestone project. J Grad Med Educ 2014;6(1 Suppl 1):129–143. 24701276 10.4300/JGME-06-01s1-07 PMC3966582

[32] Braddock CH, 3rd, Edwards KA, Hasenberg NM, Laidley TL, Levinson W. Informed decision making in outpatient practice: Time to get back to basics. JAMA 1999;282:2313–2320. 10612318 10.1001/jama.282.24.2313

[33] Braddock C, 3rd, Hudak PL, Feldman JJ, Bereknyei S, Frankel RM, Levinson W. “Surgery is certainly one good option”: Quality and time-efficiency of informed decision-making in surgery. J Bone Joint Surg Am 2008;90:1830–1838. 18762641 10.2106/JBJS.G.00840 PMC2657309

[34] Salyers MP, Matthias MS, Fukui S, et al. A coding system to measure elements of shared decision making during psychiatric visits. Psychiatr Serv 2012;63:779–784. 22854725 10.1176/appi.ps.201100496 PMC3982790

[35] Tucker Edmonds B, McKenzie F, Panoch J, Litwiller A, DiCorcia MJ. Evaluating shared decision-making in periviable counseling using objective structured clinical examinations. J Perinatol 2019;39:857–865. 30944399 10.1038/s41372-019-0366-1

[36] Tucker Edmonds B, Hoffman SM, Laitano T, et al. Evaluating shared decision making in trial of labor after cesarean counseling using objective structured clinical examinations. MedEdPORTAL 2020;16:10891. 32342013 10.15766/mep_2374-8265.10891 PMC7182044

[37] Truglio-Londrigan M, Slyer JT, Singleton JK, Worral P. A qualitative systematic review of internal and external influences on shared decision-making in all health care settings. JBI Libr Syst Rev 2012;10:4633–4646. 27820528 10.11124/jbisrir-2012-432

[38] Morley L, Cashell A. Collaboration in health care. J Med Imaging Radiat Sci 2017;48:207–216. 31047370 10.1016/j.jmir.2017.02.071

[39] Tucker Edmonds B, McKenzie F, Panoch JE, White DB, Barnato AE. A pilot study of neonatologists' decision-making roles in delivery room resuscitation counseling for periviable births. AJOB Empir Bioeth 2016;7:175–182. 27547778 10.1080/23294515.2015.1085460 PMC4990074

[40] Dehlendorf C, Krajewski C, Borrero S. Contraceptive counseling: Best practices to ensure quality communication and enable effective contraceptive use. Clin Obstet Gynecol 2014;57:659–673. 25264697 10.1097/GRF.0000000000000059 PMC4216627

[41] Rodriguez J, Abutouk M, Roque K, Sridhar A. Personalized contraceptive counseling: Helping women make the right choice. Open Access J Contracept 2016;7:89–96. 29386940 10.2147/OAJC.S81546 PMC5683162

[42] Espey E, Ogburn T, Leeman L, Singh R, Ostrom K, Schrader R. Effect of progestin compared with combined oral contraceptive pills on lactation: A randomized controlled trial. Obstet Gynecol 2012;119:5–13. 22143258 10.1097/AOG.0b013e31823dc015 PMC3586805

[43] Kelsey JJ. Hormonal contraception and lactation. J Hum Lact 1996;12:315–318. 9025449 10.1177/089033449601200419

[44] Progestin-only methods safe during lactation. Netw Res Triangle Park N C 1993;14:32–33. 12287157

[45] Gomez AM, Fuentes L, Allina A. Women or LARC first? Reproductive autonomy and the promotion of long-acting reversible contraceptive methods. Perspect Sex Reprod Health 2014;46:171–175. 24861029 10.1363/46e1614 PMC4167937

[46] Gilliam ML, Warden M, Goldstein C, Tapia B. Concerns about contraceptive side effects among young Latinas: A focus-group approach. Contraception 2004;70:299–305. 15451334 10.1016/j.contraception.2004.04.013

[47] Yee L, Simon M. The role of the social network in contraceptive decision-making among young, African American and Latina women. J Adolesc Health 2010;47:374–380. 20864007 10.1016/j.jadohealth.2010.03.014 PMC2945601

[48] Khan K, Ramachandran S. Conceptual framework for performance assessment: Competency, competence and performance in the context of assessments in healthcare—Deciphering the terminology. Med Teach 2012;34:920–928. 23039835 10.3109/0142159X.2012.722707

[49] Wallenstein J, Heron S, Santen S, Shayne P, Ander D. A core competency-based objective structured clinical examination (OSCE) can predict future resident performance. Acad Emerg Med 2010;17:S67–S71. 21199087 10.1111/j.1553-2712.2010.00894.x

