# Supplementary material for: Evaluating Shared Decision-Making in Postpartum Contraceptive Counseling Using Objective Structured Clinical Examinations
Source: Womens Health Rep (New Rochelle). 2022 Dec 26;3(1):1029–36. doi: 10.1089/whr.2022.0067 (PMC9811846; doi:10.1089/whr.2022.0067)
Supplement: Supplemental data [file Suppl_AppSA1.doc]

**Appendix A.** **Postpartum OSCE SP Profile and Instructions**

| **Your name** | Regina Wilson |
| --- | --- |
| **Presenting Situation** | Being seen by resident physician during postpartum rounds |
| **Psychosocial Profile** (Behavior, attitude, etc.) | **Background**: You just delivered your 3^rd^ daughter and are recovering well with no complaints. You are unsure which birth control is best for you. You hope to breastfeed, but had difficulty in the past. “*My* *milk never really came in”*.  **Goals & Preferences**: You want birth control and to exclusively breastfeed for 6 months. This might be your last baby unless you try again for a boy, but not for a few years. You’re bad at remembering pills and dislike needles. You failed taking the pill (*“Got pregnant with my second daughter while I was on the pill!”*) and do not want a shot every 3 months. If asked which is more important – breastfeeding or birth control – you’d say breastfeeding. You are confident that you can wait until the 6-week visit before resuming sexual intercourse. “*I can’t get pregnant if I don’t have sex, and I definitely won’t be having sex before I get on birth control. I’ve learned that lesson”*.  **Decision:** If told that progestin could potentially harm your chances at breastfeeding, you will delay getting it until the 6-week visit. If told that it would not impact breastfeeding, you will opt for the implant prior to discharge. |
| **Opening Statement** | *“Can we go over those birth control options one more time?”* |
| **History of Present Illness** | 29 years old. Delivered yesterday morning at full-term. No complications. You are feeling well. Your bleeding and pain are minimal. Baby (Annie) is doing well (she is in the nursery). |
| **Medications** | Motrin (postpartum pain control), prenatal vitamins, stool softeners |
| **Social History** | No smoking/tobacco/drug use. Live with your 2 daughters (ages 2 & 4). Boyfriend/Father of the baby is involved. Limited family in the area (just a cousin). |
| **Family Medical History** | High blood pressure and high cholesterol in mom and dad, no blood clots or bleeding problems. |
| **Additional information** | Allergies – none. Risk factors – none. Past medical history – none. |
