# Supplementary material for: Evaluating Shared Decision-Making in Postpartum Contraceptive Counseling Using Objective Structured Clinical Examinations
Source: Womens Health Rep (New Rochelle). 2022 Dec 26;3(1):1029–36. doi: 10.1089/whr.2022.0067 (PMC9811846; doi:10.1089/whr.2022.0067)
Supplement: Supplemental data [file Suppl_AppSA2.doc]

**Appendix B. Resident Directions Prior to Encounter and SP Medical Report**

**Postpartum OSCE Door Note & Instructions**

**Patient Name (age)**: Regina Wilson (29 years old)

**Setting:** Postpartum, in-hospital rounds

**Complaint**: Needs postpartum contraceptive counseling

**History of Present Illness:** Ms. Wilson is a 29 year old female G3P3003 PPD 1 s/p NSVD at 39 2/7 weeks. Her pregnancy and delivery were uncomplicated. Her medical, surgical, and social histories are unremarkable. No personal or family history of bleeding or clotting disorders. Her review of systems (ROS) is noncontributory and her exam findings are below:

**Exam**:

| Temperature = 98.2° | Respiratory Rate = 20 | Blood pressure = 114/63 | Heart Rate = 83bpm |
| --- | --- | --- | --- |
| Abdomen: soft, non-tender, fundus firm at 1 below the umbilicus | Ext: no edema, non-tender | No abnormality detected, no apparent distress | Alert and oriented, to person, place, and time |

**Instructions:** *You have 15 minutes to counsel the patient as you would in your typical practice and develop a plan of care for contraception.*
