# Supplementary material for: Evaluating Shared Decision-Making in Postpartum Contraceptive Counseling Using Objective Structured Clinical Examinations
Source: Womens Health Rep (New Rochelle). 2022 Dec 26;3(1):1029–36. doi: 10.1089/whr.2022.0067 (PMC9811846; doi:10.1089/whr.2022.0067)
Supplement: Supplemental data [file Suppl_AppSA3.doc]

**Appendix C. Postpartum Contraception OSCE Evaluation Form with Scoring Examples**

| **Element of Informed Decision Making** | **Score** | **Explanation and Example** |
| --- | --- | --- |
| **1. Discussion of the patient’s role in decision-making**  (e.g. statement that the patient can or should participate in the decision) | Absent (0) | **No indication of patient’s role in decision making:**  *“There should be a plan to prevent another pregnancy.”* |
|  | Present (1) | **Resident indicates the patient has a role:**  *“One thing that I wanted to talk to you about is contraception. Have you thought about it? I’ll talk to you about the different options and see what you want to do.”* |
|  | Complete (2) | **Resident indicates a SHARED role:**  *“I hear you wanted to talk about contraception…we can certainly talk about your options…What works best for you… and your lifestyle?”* |
| **2. Discussion of the clinical issue or nature of the decision**  (e.g. statement of what is at issue – rapid repeat pregnancy, risk of preterm labor, impact on breastfeeding, etc.) | Absent (0) | **No mention of clinical issue** |
|  | Present (1) | **Presents one side or the other:**  *“We have pretty good data information from lots of women that have been studied with these methods that all those things that I just told you about… really unlikely that they’re going to have any effect on breastfeeding”* |
|  | Complete (2) | **Requires that both sides are presented:**  “*With all of these we would only give you the progesterone-containing birth control while you’re getting started with breastfeeding. There is a small, small chance that it could make it more difficult for you to initiate breastfeeding. But that is a low risk…Benefits of not having another unplanned pregnancy are something else to consider.”* |
| **3. Does the Resident discuss alternatives**  a.) Explicit discussion of options  i. Combined hormonal contraception (OCPs/Pill, Patch, Ring)  ii. Progesterone-only contraception (the ‘mini pill’, Medroxyprogesterone/ ‘The Shot’, hormonal IUD, etonogestrel implant/ ‘The Implant’  iii. Non-hormonal contraception (Non-Hormonal IUD, condoms)  b.) Explicit discussion of delayed initiation | Absent (0) | **Only one option presented, no discussion of delayed initiation:**  *“We recommend that you get ‘The Implant’ or ‘The Shot’ before you leave the hospital.”* |
|  | Present (1) | **More than one category presented, no discussion of delayed initiation:**  *“There are several options for contraception including: OCPs, skin patches, IUDs, a vaginal ring, and The Implant.”* |
|  | Complete (2) | **Discusses at least two categories AND delayed initiation:**  *“The one that goes in the arm, we could place before you leave the hospital and any of the IUDs, we would place at your postpartum visit in four to six weeks. And if you did choose one of the IUDs, we could give you ‘The Shot’ to cover you until you get your IUD placed.”* |
| **4a. Does the Resident discuss risks of the methods?**  (e.g. venous thromboembolism, method failure, reduced milk supply) | Absent (0) | **No mention of risks** |
|  | Present (1) | **Resident discusses risk for some but not all options presented:**  *“You might be interested in ‘The Implant’ or ‘The Hormonal IUD’. ‘The Implant’ will last for 3 years. The risks of the IUD includes some irregular bleeding and some cramping.”* |
|  | Complete (2) | **Resident discusses at least one risk for each option presented:**  *“The pill must be taken every day at the same time. It can also reduce your milk supply. You might have irregular bleeding with ‘The Implant’ and ‘The Shot’ may be associated with weight gain.”* |
| **4b. Does the Resident discuss benefits of the methods?**  (e.g. efficacy, ease of use, timing of administration) | Absent (0) | **No mention of benefits** |
|  | Present (1) | **Resident discusses benefits for some but not all options presented:**  *“You might be interested in the pill or a hormonal IUD. The pill must be taken every day at the same time and may affect your breastfeeding. The IUD should not interfere with breastfeeding, may reduce menstrual bleeding, and will last 5 years.”* |
|  | Complete (2) | **Resident discussed at least 1 benefit for each option presented:**  *“You might be interested in ‘The Implant’, ‘Non-Hormonal IUD’, or ‘Hormonal IUD’. Neither should affect your breastfeeding nor require daily use.”* |
| **5. Does the Resident discuss the patient’s goal/context of decision?**  (e.g. future fertility, breastfeeding intention, prior experience with contraception, prior experience with breastfeeding, daily routine, cost, availability/access) | Absent (0) | **Resident made no effort to elicit patient’s concerns, goals, or factors:**  *“Once you hear the options available to you, you can let me know which you prefer.”* |
|  | Present (1) | **Resident asked about at least one factor:**  *“Are you thinking about having more children in the future?”* |
|  | Complete (2) | **Resident asked about 2+ factors AND inquired about timing preference:**  *“Is there anything that you’ve ever used in the past? What are your plans for your family in the future? A lot of good options that work really, really well with breastfeeding…Sometimes it can be difficult to make it to that 6-week visit.”* |
| **6. Does the Resident discuss the uncertainties associated with the decision?**  (e.g. possible, though not probable to impact breastfeeding, failure rates associated with contraception) | Absent (0) | **Resident does not mention uncertainties** |
|  | Present (1) | **Resident mentions uncertainty related to breastfeeding OR contraceptive outcomes:**  *“If you’re taking the pill exactly when you’re supposed to, it’s about 98% effective… hormonal IUDs have less than a 1% risk of getting pregnant… ‘The Shot’ is about 97-98% effective.”* |
|  | Complete (2) | **Resident mentions uncertainty related to breastfeeding AND contraceptive outcomes:**  *“Most recent studies have shown that ‘The Implant’ does not affect the milk supply… so we as OBGYNs think it is safe…99.9% effective.”* |
| **7. Does the Resident discuss the patient’s desire for input from others?** | Absent (0) | **Resident does not inquire about input from others:**  *“I know this is a lot of information. Let me know if you have any questions or need more information to help you decide.”* |
|  | Present (1) | **Resident implies that the patient may want input from others (implicit):**  *If at any point you and your partner decide you don’t want to have babies…”* |
|  | Complete (2) | **Resident asks if the patient wants input from others (explicit):**  *“Is there anyone you would like to involve in this decision?”* |
| **8. Does the Resident assess the patient’s understanding?**  (e.g. uses techniques like ask-Tell-Ask, Invite questions, teach back, | Absent (0) | **Resident does not assess patient’s understanding:**  *“I hope this information has been helpful.”* |
|  | Present (1) | **Resident invites patient to ask questions:**  *“What questions do you have right now?”* |
|  | Complete (2) | **Resident uses teach-back or ASK-Tell-Ask:**  *“Tell me what you understand about the options we discussed.”* |
| **9. Does the Resident explore the patient’s preference?**  (e.g. which way is the patient leaning?) | Absent (0) | **No mention of need for decision or inquiry about preference** |
|  | Present (1) | **Resident defers discussion of decision/preference:**  *“When I round on you tomorrow morning we can talk about this further. I can answer any more questions, and we can get a firm plan in place.”* |
|  | Complete (2) | **Resident asks about preference:**  *“Any of these sound intriguing to you? ...That was a lot of information. How are we doing?”* |
